# A terahertz in-line polarization converter based on through-via connected double layer slot structures

**DOI:** 10.1038/srep42952

**Published:** 2017-02-17

**Authors:** Jeong Min Woo, Sajid Hussain, Jae-Hyung Jang

**Affiliations:** 1 School of Electrical Engineering and Computer Science, Gwangju Institute of Science and Technology, Cheomdan-gwagiro 123, Gwangju 61005, South Korea; 2 Electrical Environment Research Center, Korea Electrotechnology Research Institute (KERI), 12, Bulmosan-ro 10Beon-gil, Changwon 51543, South Korea; 3 Research Institute of Solar and Sustainable Energies, Gwangju Institute of Science and Technology, Cheomdan-gwagiro 123, Gwangju 61005, South Korea

## Abstract

A terahertz (THz) in-line polarization converter that yields a polarization conversion ratio as high as 99.9% is demonstrated at 1 THz. It has double-layer slot structures oriented in orthogonal directions that are electrically connected by 1/8-wavelngth-long through-via holes beside the slot structures. The slots on the front metal-plane respond to the incident THz wave with polarization orthogonal to the slots and generates a circulating surface current around the slots. The surface current propagates along a pair of through-via holes that function as a two-wire transmission line. The propagating current generates a surface current around the backside slot structures oriented orthogonal to the slot structures on the front metal layer. The circulating current generates a terahertz wave polarized orthogonal to the backside slot structures and the 90° polarization conversion is completed. The re-radiating THz wave with 90° converted polarization propagates in the same direction as the incident THz wave.

Remarkable advances in terahertz (THz) technology^1^,^2^ during the last twenty years, have been observed in the area of THz devices such as sources^3^, modulators^4^,^5^, filters^6^, absorbers^7^, and polarizers^8^. As THz sources, such as photoconductive THz emitters^9^ and quantum-cascade lasers^10^, are readily available, interest in an integrated THz system has increased because of the potential applications offered by these frequencies. Even though the THz wave generated by these methods is linearly polarized, it is necessary to control the polarization in a practical THz sensor and spectroscopy systems to enhance their sensitivity and resolution^11^,^12^. Furthermore, the importance of controlling the polarization states in a single-pixel imaging system to allow a high-frame-rate was reported^13^. Polarization converters, such as wave plates, are commercially available but it is quite difficult to fabricate bulky crystal devices that require tens of wavelength thick crystals to achieve the required polarization conversion. Considering the wavelength of the THz wave, in order to develop a next generation compact and light-weight THz system, it is necessary to design polarization control devices whose device dimensions are much smaller than the operating wavelength of the THz wave. At lower frequencies in a microwave or millimeter wave regime, polarization control may be achieved with the help of antenna networks^14^. Researchers have also sought to realize a polarization converter in sub-THz frequencies, by using a metallic waveguide^15^ and a quasi-optical method^16^. Although extensive efforts have been devoted to develop a powerful polarization convertor, a feasible solution to control the polarization state has yet to be found. Meanwhile, artificially structured subwavelength-scale metamaterials^17^,^18^,^19^,^20^,^21^ are highly attractive because of their exotic electromagnetic properties that cannot be achieved with natural materials.

A metamaterial structure responds to a terahertz wave based on its geometric structure rather than material composition, and its thickness is typically on a subwavelength scale. It manipulates and controls the electromagnetic wave in a range from microwave to THz frequencies. Furthermore, polarization conversion has been achieved in terahertz frequencies by using extrinsic chirality of the metamaterials^22^,^23^,^24^. This metamaterial based polarization converter research was originally based on circular birefringence, but it suffered from a high insertion loss (9.1 dB) and low polarization conversion angle (12°). To improve the performance, a three-dimensional mesh and a multilayer metamaterial structure have been proposed^25^,^26^. Those devices rotated the polarization angle around 45° and 90°, respectively, but the improved polarization conversion ratio (97.8%) was based on Fabry-Perot resonance by a continuous wave source and still required a complex fabrication process. A metamaterial structure combined with a ground plane was also investigated for a reflection type polarization converter^27^,^28^,^29^. They achieved a polarization conversion angle up to 90° with a low insertion loss (2 dB) but they had a lower polarization conversion ratio (>85%). As many THz applications require a polarization converter to operate in a transmission mode, there is a need for an inline device with a higher polarization conversion ratio while maintaining the benefits of a reflectance-type metamaterial structure.

This paper demonstrates a highly efficient, inline polarization converter that operates in a THz frequency range and uses a three-dimensional metamaterial structure. It rotates the polarization angle of an incident terahertz wave by 90° while maintaining a high polarization conversion ratio as well as the propagation direction. The polarization converter presented in this paper is based on double-layer shorted metallic apertures separated by a thin substrate. It overcomes previously introduced limitations of bulk crystal and reflectance-type metamaterial devices and can readily be used in any THz system as an inline device. Here, the sub-wavelength apertures in thin metallic films are of particular importance because they can be designed to resonate up to the terahertz frequency range. These rectangular slot apertures have been utilized in lower frequencies since 1970 after the numerical explanation of Chen^30^, and they have been incorporated in slot antennas^31^ and filters^32^. By electrically connecting two metallic slot apertures that receive and transmit orthogonally polarized waves, highly linear polarization conversion has been achieved.

## Results

### Design of the unit cell

**Figure 1:**
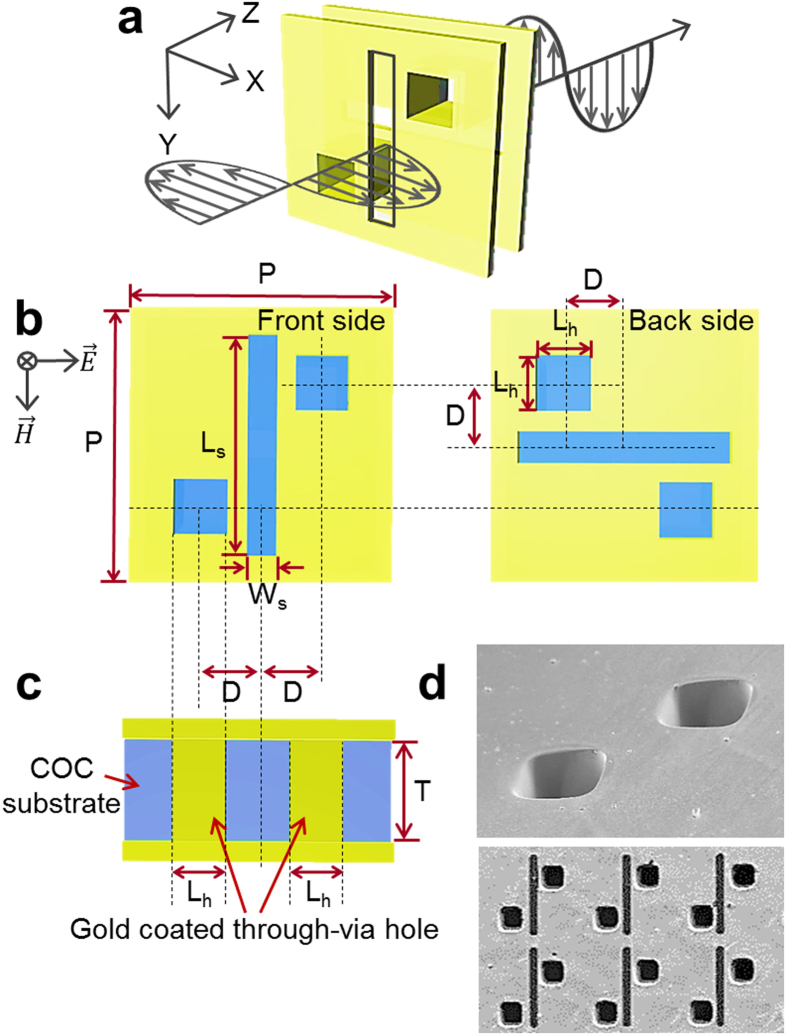
Design of the terahertz polarization converter. (**a**) Schematic view of a polarization converter consisting of two orthogonally oriented metallic apertures and gold coated through-via holes. (**b**) Geometry and dimensions of the unit cell of the top and bottom apertures and though-via holes: the length of slot L_s_ = 117 μm, the width of slot W_s_ = 15 μm, the length of hole L_h_ = 40 μm, distance D = 31 μm. The square unit cell has periodicity, P, of 134 μm to form a planar array of 5.0 × 5.0 mm^2^. (**c**) Side view of the polarization converter; the thickness, T, of the COC film is 40 μm. Two metallic layers are shorted by a pair of gold coated through-via holes. (**d**) SEM images of the through-via holes (top) and slot pattern (bottom) structures.

**Figure 2:**
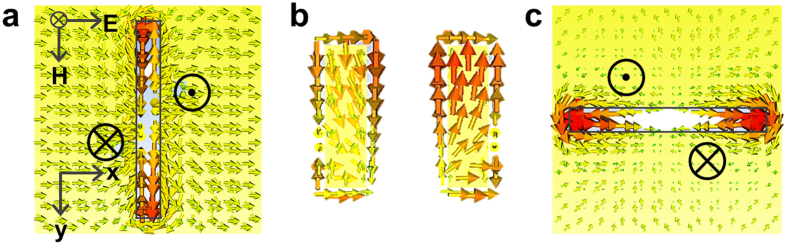
Numerical characterizations of the polarization conversion. Simulated surface current (J_m_) distributions **(a)** on the front-side aperture, **(b**) on the sidewall of though-via holes and **(c)** on the back-side aperture at frequency of 1.0 THz. The Ⓧ and ʘ marks on the aperture denote surface current flowing in and out through the through-via holes. For the simulation study, through-via holes of 5 × 5 μm^2^ are considered because clearer current direction could be obtained with these smaller through-via holes.

Figure 1a shows a schematic diagram of a unit cell of the polarization converter. Metallic apertures are perforated into a thin gold film deposited on both sides of the 40-μm-thick cyclo-olefin-copolymer (COC) film. Two metallic layers, whose apertures are arrayed in orthogonal directions, are connected with each other at diagonal locations using gold-coated through-via holes as shown in Fig. 1b–d. (see Methods and Supplementary Figs 1 and 2). The transmission response was not affected by the size of the through-via hole, as shown in Supplementary Fig. 3. The position of the through-via hole structure has an influence on the co-polarized transmission, t_xx_. As the distance between the via hole and slot structures, D, is increased, t_xx_ becomes larger. D is kept as small as possible within the fabrication margin. As shown in Fig. 1a, the x-polarized electric field is converted to y-polarization when the incident electromagnetic wave propagates through the device. An x-polarized incident terahertz plane wave propagating in the z-direction is defined as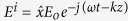. The incident THz electromagnetic wave generates surface current around the aperture on the front-side metal layer. The highest surface current density is observed at the edge of the slot aperture, whereas the highest electric field is observed at the center of the aperture structure. As shown in Fig. 2a, the surface current is induced along the slot aperture, and these surface currents are then transferred to the back-side apertures, which are oriented in the perpendicular direction, through the through-via holes. The simulated results of a single layer slot aperture without the through-via hole structure are given in Supplementary Fig. 4. As shown in Fig. 2b, a pair of through-via holes acts as a short parallel-conductor transmission line. The magnitude of the surface current on each wire is the same whereas the direction is opposite. As a result, as presented in Fig. 2c, a surface current is induced at the back-side aperture. The back-side slot aperture with a 90° rotated angle radiates 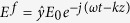 in the far-field. The front-side metamaterial plane acts as a receiver that responds to the incoming x-polarized THz radiation, while the back-side metamaterial plane acts as a transmitter radiating a y-polarized THz wave. Thus, the proposed three-dimensional metamaterial structure behaves as an effective chiral medium and induces polarization conversion at the resonance frequency.

### Effect of the through-via hole structure

**Figure 3:**
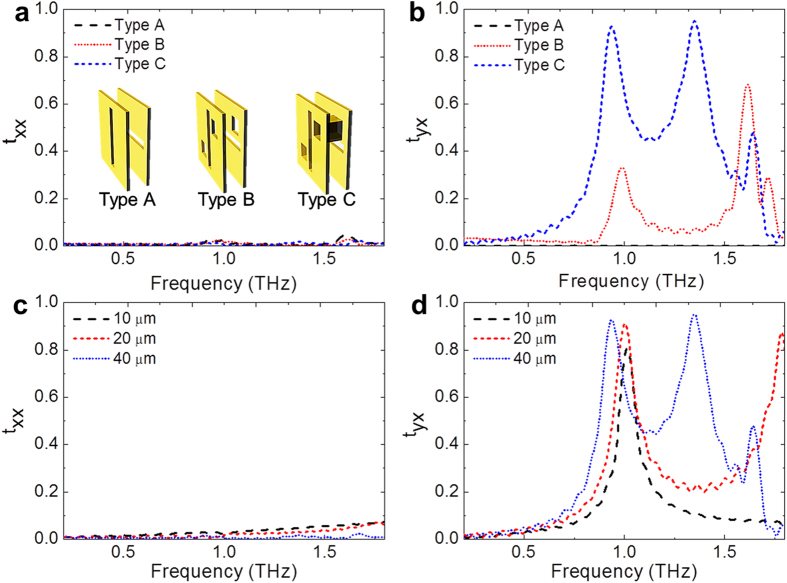
Polarization conversion characteristics of the three different structures and the effect of the substrate thickness. **(a)** Co-polarized transmission, t_*xx*_ and **(b)** 90° polarization converted transmission, t_*yx*_ for Type A (orthogonal oriented slot structure), Type B (orthogonal oriented slot structure with rectangular-hole patterns), and Type C (orthogonal oriented slot structure electrically connected by rectangular-hole patterns). Spectral responses of the polarization converters with substrates having different thicknesses (10 μm, 20 μm, and 40 μm) are calculated for **(c)** co-polarized transmission, t_*xx*_ and **(d)** 90° polarization converted transmission, t_yx_.

To study the effect of the through-via hole structure, three different types of metamaterial structures are considered as shown in Fig. 3, and the size of the through via-hole, 40 × 40 μm^2^, follows its size in the fabricated converter. The type A structure is a pair of metal planes with orthogonally oriented slot apertures without through-via holes. The type B structure is generated by adding additional through-via holes to type A. Then type C is generated by coating a conducting layer at the surface of the through-via holes, as shown in the inset of Fig. 3a. The x-polarized electromagnetic wave is incident on the front side of the metamaterial structure, which consists of slots arranged along the y direction. The type A structure completely blocks the x-polarized incoming wave and does not allow any transmission. By perforating rectangular-hole patterns on the structure (Type B), weak polarization conversion was obtained by coupling between the front and back side planes at 1.0 THz, as shown in Fig. 3a and b. For the type C structure in which the slot apertures on both sides are electrically connected by gold-coated through-via hole structures, polarization rotation occurred at around 1.0 THz, and the transmitted co-polarization (t_xx_) was also greatly suppressed compared with that of Type B. Denoting the transmitted x-polarization and y- polarization as t_xx_ and t_yx_ for the incoming x-polarized wave, the polarization conversion ratio can be defined as 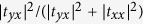, where t_yx_ corresponds to the transmission of 90° rotated polarization. The polarization conversion ratio of the Type B structure was 99.1%. For Type C, the polarization conversion ratio was greatly enhanced to 99.9%.

The substrate, which supports the metallic apertures must be a good insulator and should offer low insertion-loss in terahertz frequencies. Substrates such as polyethylene and polyester are frequently used to make wire grid type polarizers^19^,^20^ because of their higher isolation and low loss at THz frequencies. The 40-μm-thick-COC film also exhibits high transmission up to 90% at 1.0 THz. This COC film was cured in a nitrogen atmosphere to avoid material loss at the front and back side apertures. (see Method and Supplementary Fig. 5). As shown in Fig. 3c and d, the substrate thickness is critical to achieve good spectral response. When longer through-via holes were made, both the first and second resonance frequencies were redshifted. The second resonance frequency, which originates from the through-via hole structure, shows larger frequency shifting with length variations. On the other hand, decreasing the substrate thickness hinders the correct operation of the polarization converter due to the increased evanescent coupling between the front and back side metallic slot apertures. This disturbs the formation of the surface current loop at the back-side slot aperture, which decreases the radiated electric field strength (see Supplementary Fig. 6).

### Measurement of the polarization conversion

**Figure 4:**
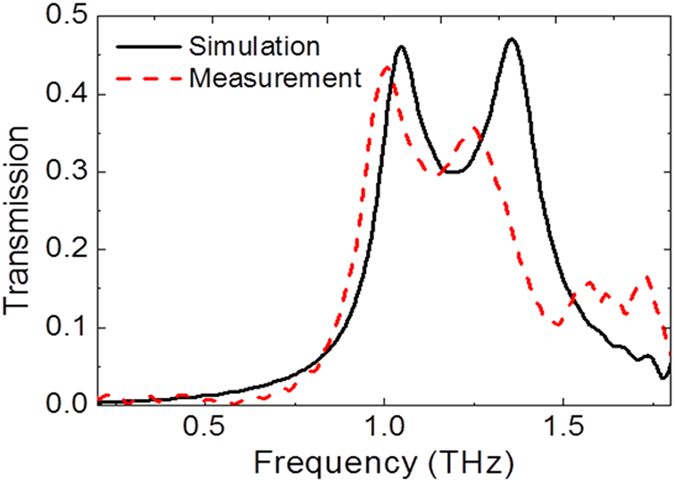
Comparison between simulated and measured transmission characteristics of a single polarization converter rotated at 45°. Transmission spectra of the simulated and experimental results of a single polarization converter.

A conventional terahertz time-domain spectroscopy system^33^ was used for the characterization of the fabricated devices. Because the detector, receiving x-polarization only, cannot measure cross-polarization in our TDS measurement setup^34^, the transmission spectra of a single device were measured by rotating it in the measurement setup. The maximum transmission was observed at an angle of 45° as shown in Table 1, and schematic views of rotated samples are shown in Supplementary Fig. 7. The transmission level increased when the device rotation angle increased up to 45°. As shown in Fig. 4, the measurement and simulation results exhibit similar transmission levels at a frequency of around 1.0 THz. The transmission levels of the 45°-rotated device were 0.43 and 0.46 for the measurement and the simulation, respectively. As the results for Type C in Fig. 3 show, the polarization converted transmission level of the 45° angle-rotated device is half that of the un-rotated device. As the device was rotated, coupling of the x-polarized incident electric field was reduced by half. This high polarization selectivity is a typical characteristic of the slot structures^35^.

**Figure 5:**
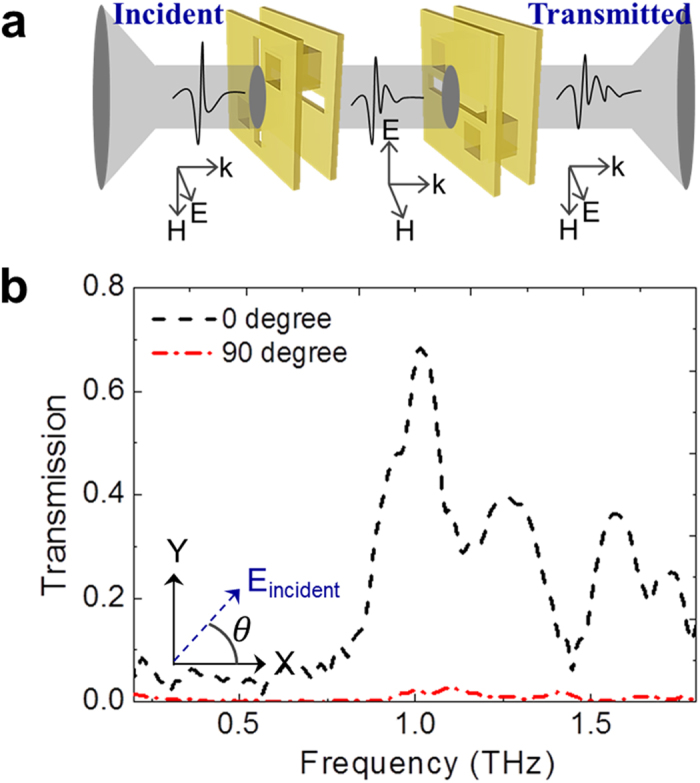
Measurements of the cascaded polarization converter. **(a)** Two polarization converters are placed together to recover the initial polarization of the incident terahertz wave. **(b)** Transmission characteristics of the cascaded dual polarization converters. Two polarization converters are orthogonally oriented. The inset shows the rotation angle of the combined two polarization converters. Transmission characteristics are shown for the polarization rotated incident wave with rotation angles of 0° and 90°. Less than 0.1% co-polarized transmission is observed.

In order to further verify the polarization conversion characteristics, transmission measurements were carried out for the cascaded devices where two orthogonally oriented devices are separated by 100 μm. The experimental setup is shown in Fig. 5a. For the cascaded devices, the polarization conversions were made twice to recover the incident polarization. Resonances at 1.0 THz and 1.3 THz were observed, as shown in Fig. 5b. The transmission level of the 0° angle-rotated device was 0.68 at 1.0 THz, whereas the 90° angle-rotated device had a transmission level of 0.02. The rotation angle is oriented at an angle 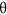 relative to the x-axis. As the rotation angle of the device increased, the transmission level decreased to almost zero, but no resonance frequency shift occurred. The primary resonance originated from the individual aperture and its frequency depends on the substrate thickness^36^, metal thickness^37^, periodicity, and the geometry of the aperture. The second resonance observed at 1.3 THz depends on the length of the through-via holes where the connecting wires are shorting the two metallic layers (see Fig. 3d and Supplementary Fig. 3). Although the second resonance frequency shifted with the length variation of the through-via hole structure, the primary resonance intensity and frequency did not change. The polarization conversion ratio is calculated to be as high as 99.9% at the resonant frequency. The suppressed x-polarized transmitted wave greatly enhances the polarization conversion overall. The insertion loss of the device is 0.7 dB according to the simulation, but the fabricated device exhibits much higher insertion loss of 3.3 dB. This discrepancy may be ascribed to imperfect fabrication during the pattern transfer process. Nevertheless, the measured polarization conversion ratio is the highest value ever reported^25^ according to Table 2, where the performance of existing polarization converters is compared.

## Discussion

The high polarization conversion ratio was achieved by orthogonally oriented three-dimensional metallic slot apertures that are electrically connected by through-via holes. The through-via holes play the main role in realizing a high polarization conversion ratio of up to 99.9%. The 90° polarization conversion was achieved at broadband from 0.3 to 1.4 THz. The primary resonance (1.0 THz) frequency is determined by the dimensions of the slot apertures, whereas the second resonance is related with the configuration of the through-via hole structures that are currently under investigation. The polarization converter also demonstrates in-line polarization conversion with only a 1/8-wavelength-thickness. The demonstrated polarization converter will be useful in many spectroscopy^38^,^39^,^40^ and imaging systems^13^,^41^. By using the polarization converter, two orthogonal polarizations can be obtained from a linearly polarized THz source. When the obtained polarization diversity is employed in a THz imaging system, a clearer reflection image can be reconstructed by combining the individual reflection images of the polarization sensitive objects.

## Methods

### Device fabrication

The fabrication of the device starts with bare silicon. An engraved square-pattern was formed on top of the silicon by conventional photolithography and a deep-dry etching process. This master stamp was coated with polydimethylsiloxane (PDMS), which has excellent elasticity. The diluted PDMS (Sylgard 184) was cured on a hot plate at 80 °C for 1 hr. The PDMS was peeled off after cooling, and the PDMS stamp was applied again to a cyclo-olefin copolymer (COC) that is coated on 50-nm-thick SiO_2_ deposited on glass substrate. The PDMS-stamp/COC/SiO_2_/glass sample was cured in an oven at 80 °C for 1 hr 40 minutes in a nitrogen atmosphere. This COC (MR-I T85–5.0 from Micro Resist Technology) layer exhibits higher absorbance when it is cured in an uncontrolled atmosphere (see Supplementary Fig. 5). To detach the stamped COC-layer without scratching the surface of the glass substrate, the sample was dipped in buffered oxide etch (BOE) for 2 hours. A thin SiO_2_ layer, sandwiched between the COC-layer and glass, was utilized as a sacrificial layer. After the COC-layer was detached, a thin COC-layer remained at the bottom of the stamped region. The sample was flipped over and reactive-ion etching (RIE) was performed to complete the perforating process. Next, the film was loaded in e-beam evaporation. The Ti/Au (20/200 nm) metallization was evaporated twice at the front and back sides of the sample. The sample was loaded on a 30° tilted holder for conformal coating of a gold layer at the sidewalls of the through-via holes. The slot patterning was performed by conventional optical lithography and a subsequent wet etching process with a mixture of nitric acid and hydrochloric acid (50 ml: 150 ml), and BOE.

### Numerical simulation

An electromagnetic simulation was performed by commercial software (HFSS). Within a waveguide model, the plane wave was placed at the front side and a probe was located at the opposite side to detect the electromagnetic response of the devices. Boundary conditions of a perfect electrical conductor (PEC) were imposed at the top and bottom, and boundary conditions of a perfect magnetic conductor (PMC) were imposed at the left and right.

### Terahertz time-domain spectroscopy

The THz time domain spectroscopy (THz-TDS) was performed by using a TPS 3000 (Teraview).

## Additional Information

**How to cite this article**: Woo, J. M. *et al*. A terahertz in-line polarization converter based on through-via connected double layer slot structures. *Sci. Rep.* **7**, 42952; doi: 10.1038/srep42952 (2017).

**Publisher's note:** Springer Nature remains neutral with regard to jurisdictional claims in published maps and institutional affiliations.

## Supplementary Material

Supplementary Information

## Figures and Tables

**Table 1:** Measured transmission characteristics of a single polarization converter.

Rotation angle	0°	45°	90°
Normalized transmission	0.019	1	0.028

**Table 2:** Performance comparison with previously reported THz polarization converters.

Device Structures	Operating frequency range	Conversion angle	Polarization conversion ratio		Insertion loss	Ref
Meta-surface + Backside metal plane	0.7~1.9 THz	90°	Transmission type	>97.8% (max.99.5%)	0.9 dB	28
			Reflection type	>85% (max.99%)	0.4 dB	
Two displaced layers of chiral patterns	14~17 GHz	90°	—		—	27
	1.0 THz	90°	97.8%		6.3 dB	25
	0.4 THz, 1.2 THz	1.5°	—		—	22
Three-dimensional structure	6~14 GHz	45°	—		—	26
	1.0 THz	90°	**99.9%**		3.3 dB	This work
