# Supplementary material for: A terahertz in-line polarization converter based on through-via connected double layer slot structures
Source: Sci Rep. 2017 Feb 17;7:42952. doi: 10.1038/srep42952 (PMC5314401; doi:10.1038/srep42952)
Supplement: Supplementary Information [file srep42952-s1.doc]

# **A terahertz in-line polarization converter based on through-via connected double layer slot structures**

Jeong Min Woo^1,2^, Sajid Hussain^1^, and Jae-Hyung Jang^1,3*^

**Supplementary Figures**


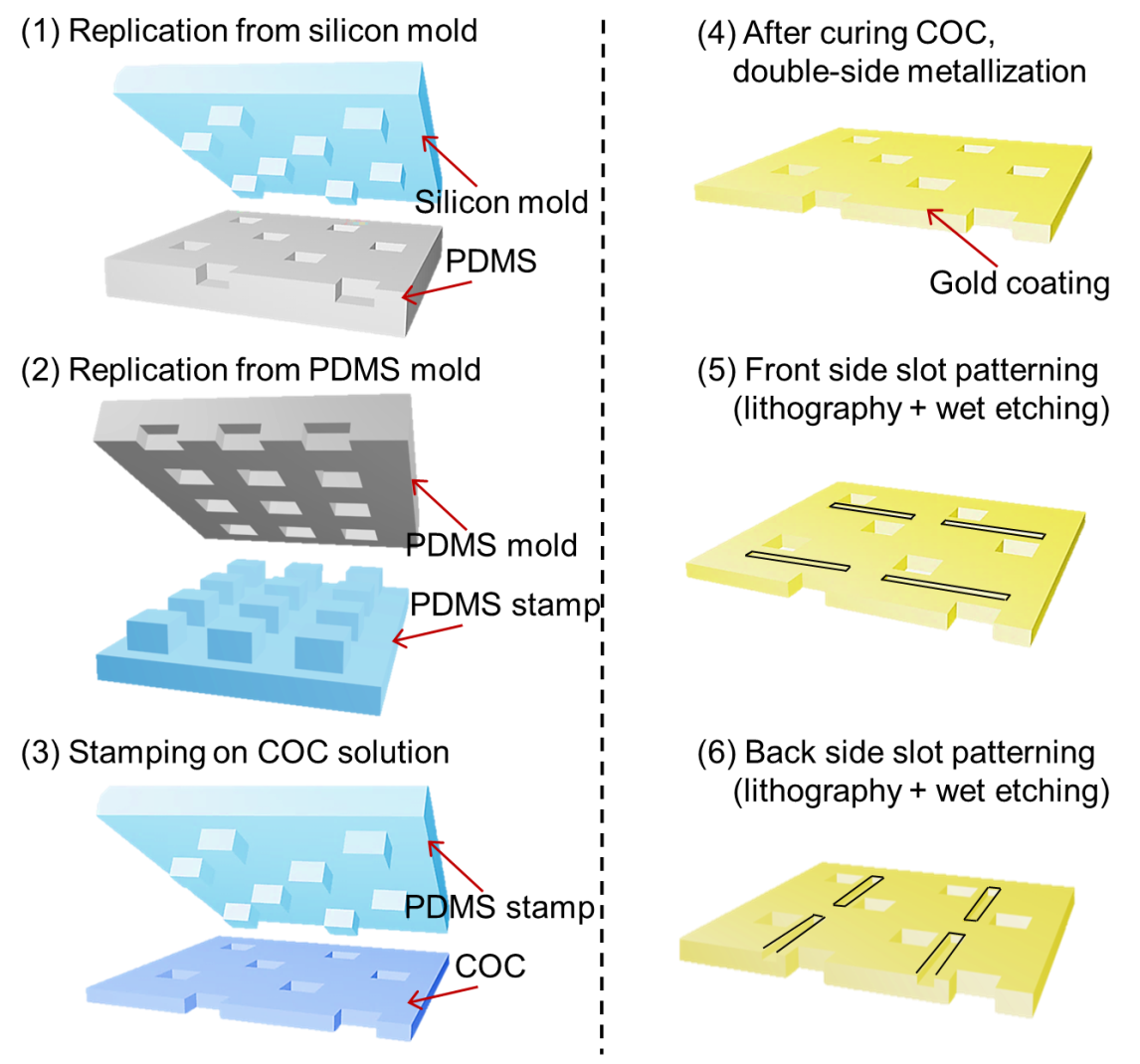


**Supplementary Figure 1| Fabrication process of the polarization converter.** The fabrication process of the polarization converter involves the following steps: (1) The master silicon mold was fabricated by conventional photolithography and a deep dry etching process. The diluted PDMS solution was coated to replicate the structure from the silicon mold, and the PDMS was peeled off after cooling. (2) The diluted PDMS solution was coated to replicate the structure from the PDMS mold, and the PDMS was peeled off after cooling. (3) The PDMS stamp was applied again to a cyclo-olefin copolymer (COC) coated 50 nm-thick-SiO_2_ deposited glass substrate. (4) After curing, COC film was detached from the glass substrate by etching sacrificial layer of SiO_2_ with BOE solution. Subsequently, gold was evaporated twice at front and back-side of the sample. (5) The slot patterning was performed by conventional optical lithography and a wet etching process. (6) Same process was performed on the back-side to pattern slot structures arranged in the orthogonal direction.


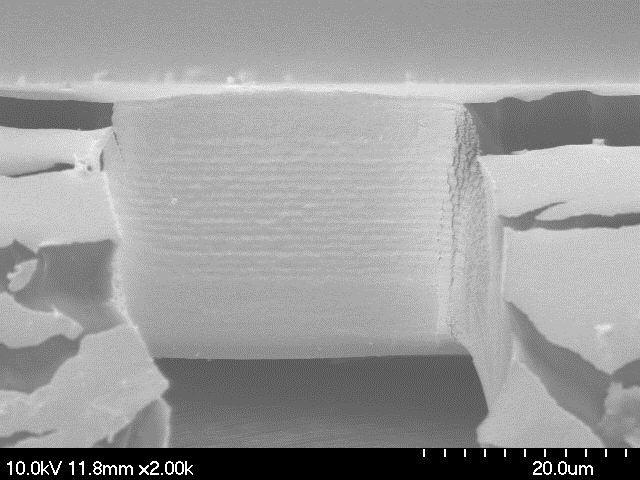


**Supplementary Figure 2| SEM picture of the cross-section of the through-via hole structure.** It is clearly shown that the COC film was completely punched through by the stamping method and the sidewall of the through-via hole structure is coated with gold. Part of the COC film was destroyed during the scribing process to obtain the cross sectional view of the through-via holes. Despite the surface roughness (SWR) at the sidewall, the scattering loss due to the SWR is negligible considering that the degree of SWR and the length of the through-via holes are much smaller than the wavelength of interest.

**
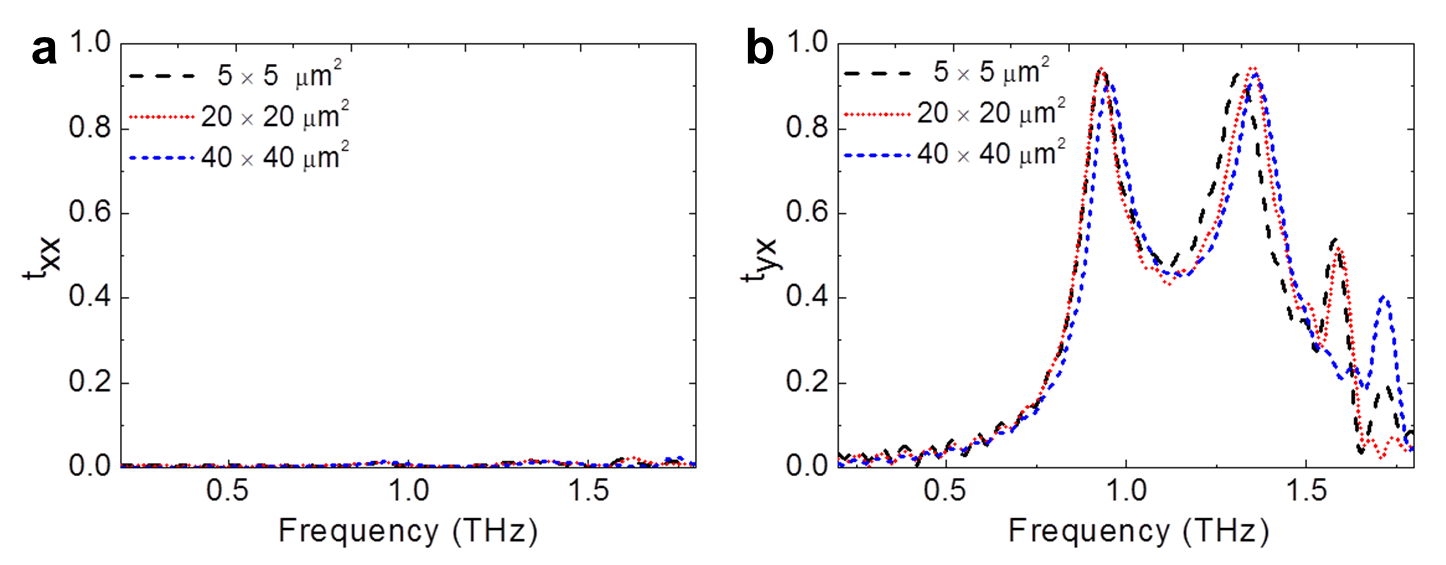
**

**Supplementary Figure 3| Simulated transmission spectra of the devices with different through-via hole structures sizes. (a)** Co-polarized transmission (t*_xx_*) and **(b)** polarization converted transmission (t*_yx_*). Even though the size of the through-via hole increases, the transmittance levels of both first and second resonance frequency does not change. The length variations of the through-via hole structure is dominant factor of the second resonance frequency shifting.


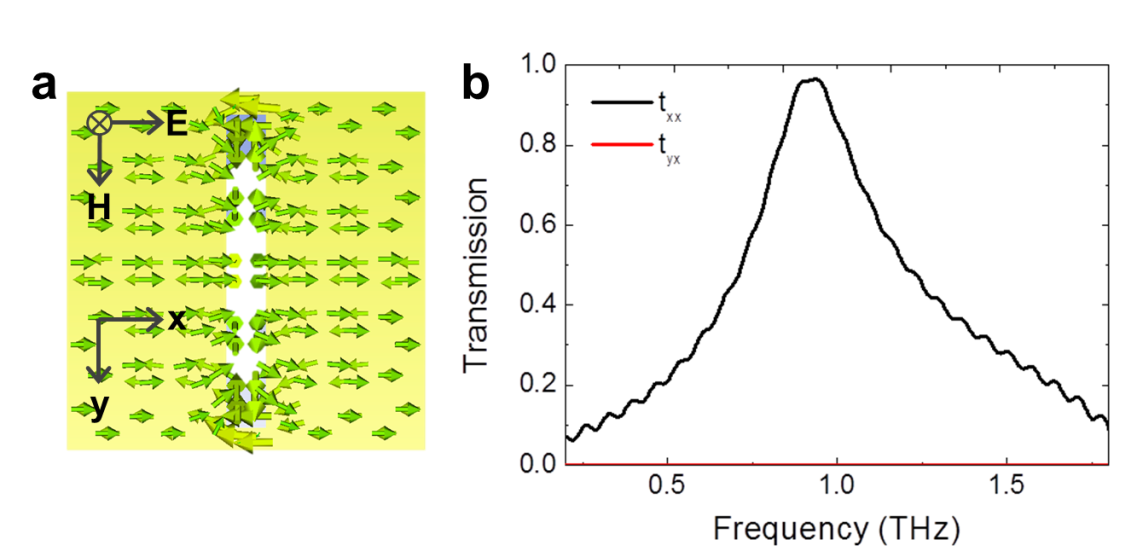


**Supplementary Figure 4| Electromagnetic simulation of the single layer slot aperture.** The transmission and surface current distribution of the single layer slot aperture are simulated. **(a)** The highest surface current density is observed at the edge of the slot aperture, whereas the highest electric field is observed at the center of the aperture structure. It is similar to the simulation results of front-side of polarization converter with through-via hole structure. **(b)** The co-polarized transmission (t*_xx_*) shows bandpass characteristic with maximum transmission level around 1 THz, whereas polarization converted transmission (t*_yx_*) is not observed.


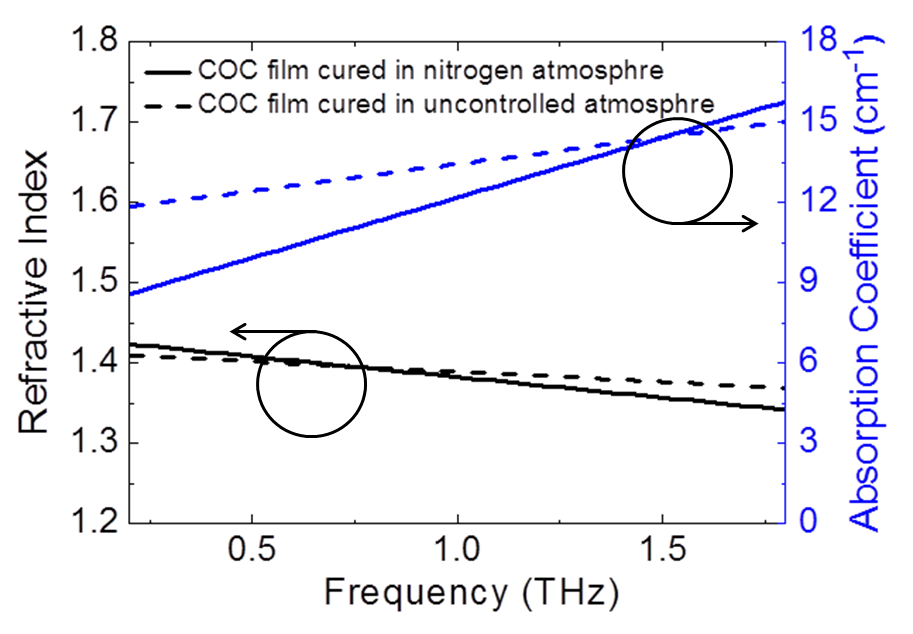


**Supplementary Figure 5| Comparison of the electromagnetic material properties of the COC films cured in nitrogen and an uncontrolled atmosphere.** The refractive index and absorption coefficient of COC film cured in nitrogen and an uncontrolled atmosphere are compared. At 1.0 THz, the refractive index of COC film cured in nitrogen and an uncontrolled atmosphere were 1.38 and 1.39, respectively. The absorption coefficients of COC film cured in nitrogen and an uncontrolled atmosphere are 12.2 and 13.4, respectively. The nitrogen atmosphere effectively prevents the formation of bonding between oxygen and polymers, thereby, making the COC film more transparent in the THz range.


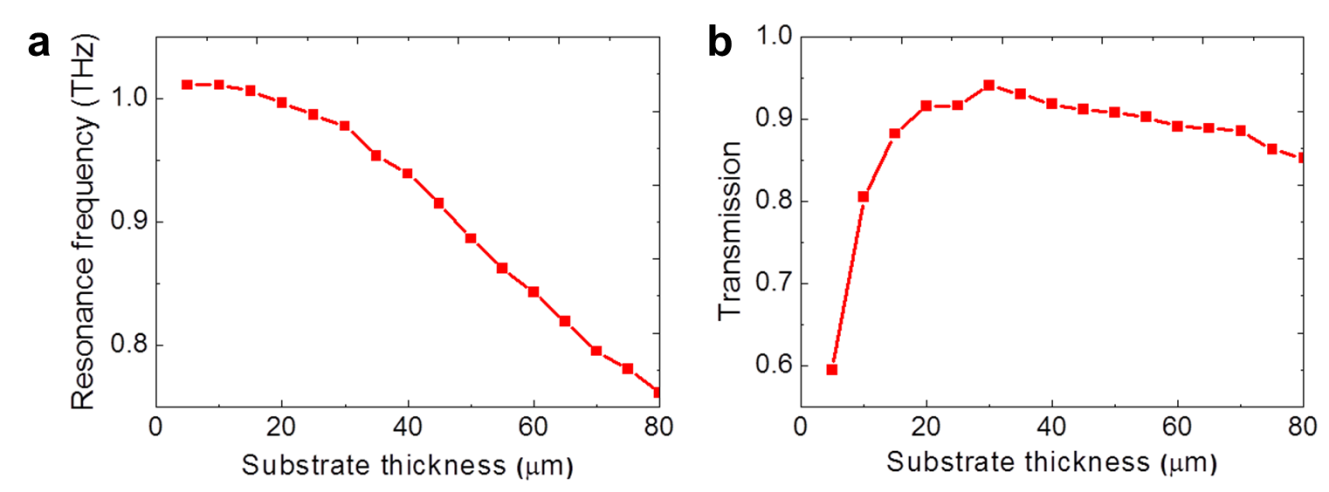


**Supplementary Figure 6| Simulated primary resonance frequency and transmission level with respect to the thickness of the COC substrate.** The performances of the polarization converters having various substrate thicknesses were simulated. **(a)** Resonance frequency shift and **(b)** changes in transmission level are observed. As the substrate gets thicker than 30 μm, the primary resonance frequency begins to shift rapidly. The transmission level at the primary resonance frequency is highest for the 30-μm-thick substrate.


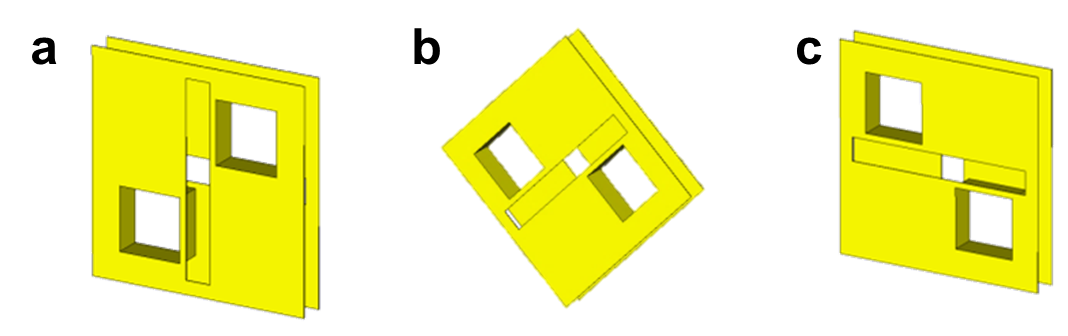


**Supplementary Figure 7| Schematic views of rotated samples.** The performance of the polarization converters have been measured and compared in **Table 1** with following rotation angles: **(a)** 0°, **(b)** 45°, and **(c)** 90°. As shown in the **Table 1**, the 45°-rotated polarization converter has shown maximum transmission when it is compared with others.
